# Urine-based testing for Chlamydia trachomatis among young adults in a population-based survey in Croatia: Feasibility and prevalence

**DOI:** 10.1186/1471-2458-11-230

**Published:** 2011-04-14

**Authors:** Ivana Božičević, Ivana Grgić, Snježana Židovec-Lepej, Jurja-Ivana Čakalo, Sanja Belak-Kovačević, Aleksandar Štulhofer, Josip Begovac

**Affiliations:** 1 WHO Collaborating Centre for Capacity Development in HIV Surveillance; School of Medicine, Zagreb, Croatia; 2 University Hospital for Infectious Diseases, Zagreb, Croatia; 3 Faculty of Humanities and Social Sciences, Zagreb, Croatia

## Abstract

**Background:**

We assessed the feasibility of collecting urine samples for testing on genital *Chlamydia trachomatis *infection in a population-based survey, and prevalence of this infection among young people aged 18-25 in Croatia. In Croatia, as in the other countries of Eastern Europe, there is a lack of data on prevalence of *C trachomatis *in the general population, including young adults.

**Methods:**

We sampled participants using a nationally representative, multi-stage stratified probability sample of young men and women. Detection of *C trachomatis *DNA in urine samples was performed by using a real-time PCR assay COBAS^® ^TaqMan^® ^CT Test, v2.0.

**Results:**

Overall, 1005 young adults participated in the behavioural part of the survey, and 27.9% men and 37.5% women who were sexually experienced agreed to provide urine samples for testing on *C trachomatis*. Using multivariate analysis, women were significantly more likely to provide urine samples than men (aOR = 1.53, 95% CI 1.14-2.06) as were those who reported no condom use at last intercourse (aOR = 1.95, 95% CI 1.44-2.62). Prevalence of *C trachomatis *infection among those who were sexually experienced was 7.3% in men and 5.3% in women.

**Conclusions:**

Population-based surveys that use probabilistic sampling are a feasible way to obtain population estimates of *C trachomatis *prevalence among young adults in Croatia, but it is challenging to obtain an adequate response rate. The prevalence of *C trachomatis *among young adults in Croatia found in this study was higher than that found in other European countries with similar survey response rates.

## Background

*Chlamydia trachomatis *is the most common bacterial sexually transmitted infection (STI) in Croatia. In 2008, 11 cases of *Neisseria gonorrhoeae*, 31 cases of syphilis and 553 cases of genital *C trachomatis *infection were reported [1]. However, those figures are based on case reporting, and are believed to substantially underestimate true incidence. *C trachomatis *case rates in Croatia ranged from 7.04/100,000 in 1999 to 9.11/100,000 in 2007, compared to 3.99/100,000 to 10.04/100,000 in Slovenia, and 96.32/100,000 to 200.26/100,000 in the United Kingdom [1]. In Croatia, as in the other countries of Eastern Europe, there is a lack of data on prevalence of *C trachomatis *in the general population, including young adults [2]. The focus of STI surveillance in Eastern Europe has been mainly on universal case reporting and interpretation of trends in aggregated national rates of STIs: this obfuscates variations in burden of infections among different demographically and behaviourally defined groups in the population. Although compulsory in most countries, reporting of bacterial STIs in Eastern Europe is mostly done by public institutions and tends to be incomplete [3].

The effectiveness of case reporting systems for *C trachomatis *depends on screening policies and health-seeking behaviour, service utilisation and reporting practices of STI service providers. Population-based studies on prevalence of curable bacterial STIs, including *C trachomatis*, are needed to make evidence-based decisions on whom to screen [4]. Such studies have a crucial role in surveillance of STIs as they rely on probability-based sampling techniques, which enables them to obtain robust and comprehensive data on demographic and behavioural risk factors for infections [5].

This study aimed to estimate the HIV and STI-related risk behaviors among young adults aged 18-25 in Croatia in 2010 and to compare these results with the results of a similar survey that was conducted in 2005 [6,7]. In addition, the study assessed the feasibility of collecting urine samples to test for and determine the prevalence of *C trachomatis *infection. Data on sexual behaviours and comparison with results of the survey carried out in 2005 will be described elsewhere.

## Methods

### Sampling

This study was carried out in a nationally representative, multi-stage stratified probability sample (n = 1005) of young men and women aged 18-25 years. Similar multi-stage cluster-based households surveys were used to measure sexual behaviours and *C trachmatis *prevalence in Great Britain and Slovenia [8,9]. Multi-stage stratified survey design is recommended by the World Health Organization in order to obtain nationally representative estimates of HIV and STIs [10]. The sample size calculation for this cross-sectional survey was based on the +/- 10% change in condom use at most recent sexual intercourse from the baseline of 55% observed in the 2005 national probability sample of the same population [6]. As no reliable population-based data existed on the prevalence of *C trachomatis *among Croatian young adults, this biological indicator was not employed in the sample size calculations.

Data were collected in February and March 2010. We stratified the sample by county, size of settlement (≤2000 persons; 2001-10 000; 10 001-100 000; >100 001), age and sex. We based the sampling frame on the results of the most recent census, which was conducted in 2001. We randomly selected 106 sampling points (settlements) from all 21 counties of Croatia proportional to the population size. Following a detailed procedure for randomised selection of households, 9-10 participants were interviewed at each sampling point. Where there was more than one resident aged 18-25 years in one household, we selected the young adult who had the most recent birthday to participate in the survey.

### Questionnaire

The questionnaire consisted of two parts. A trained interviewer administered the first part face-to-face; this included questions on demographic and socio-economic characteristics of respondents, knowledge and attitudes towards HIV/AIDS, and beliefs about condoms and condom use. The second part of the questionnaire was self-administered and contained questions related to sexual behaviours and experiences. The questionnaire was modified from the survey conducted in 2005 [6]. We pilot tested this modified questionnaire for comprehensiveness and completion time among 235 students from four metropolitan secondary schools in 2009.

### Interviews and specimen collection

A majority of interviewers were women between 25 and 35 years of age, who had considerable experience in interviewing for one or more national research companies. All interviewers received an additional six hours of training focused on interviewing young adults on sensitive topics and collection of biological samples. All participants were interviewed in their homes, after giving informed consent. Consent for providing urine samples was administered separately at which time information and guidance on how to take urine samples was provided. All individuals who gave urine received an information sheet with a unique sample code and a toll-free number to call for a test result. As no names or other personal identifiers were collected, participants were instructed to call to obtain their test results. All data collection was anonymous and confidential.

Phone control of the field work was carried out by study co-ordinators on 30% of the households included in the survey. All study procedures were approved by the Ethical Review Board of the Faculty of Humanities and Social Sciences, University of Zagreb and the Ethical Board of the University Hospital for Infectious Diseases "Dr. Fran Mihaljević" in Zagreb.

Detection of *C trachomatis *DNA in urine samples was performed using a real-time PCR assay COBAS^® ^TaqMan^® ^CT Test, v2.0 (Roche Molecular Systems, Branchburg, NJ, USA). Following manual DNA extraction, we performed real-time PCR using COBAS^® ^TaqMan^® ^48 Analyzer (Roche Molecular Systems, Branchburg, NJ, USA), as recommended by the manufacturer. This real-time PCR assay employs two sets of probes and primers for amplification of two separate target sequences, one for the *C trachomatis *cryptic plasmid and other for the *ompA *gene of the *C trachomatis *chromosome, which ensures the detection of all *C trachomatis *strains (including the new Swedish variant) [11].

### Analyses

We compared factors associated with providing urine among those who had previousely had sexual intercourse using bivariate and multivariate logistic regression analyses. The following socio-demographic and behavioural variables were considered potential correlates with respect to the likelihood of providing urine and the prevalence of *C trachomatis *infection: gender, self-estimated socio-economic status, living with both parents until 18 years of age, having obtained information on HIV/AIDS at school, age of first sexual intercourse, number of sexual partners in the past 12 months and in the lifetime, use of condoms at last intercourse and ever having a concurrent partnership. Results are presented as odds ratios (ORs) with 95% confidence intervals (95% CI). Variables associated with willingness to provide urine at the level of p < 0.2 on bivariate analysis were included in the multivariate logistic regression model. The cut-off for considering a result to be statistically significant in the multivariate analysis was set up at p = 0.05.

Analysis of data was performed using STATA 8.0 [12]. Participants with missing values were excluded from the analyses. Due to the small number of individuals testing positive for *C trachomatis *infection, multivariate regression analysis was not conducted and we compared the prevalence of *C trachomatis *infection across socio-demographic and behavioural variables with chi square tests and Fisher exact tests. Fisher's exact test was used when the expected value in any of the cells of a contingency table was below 5.

## Results

Overall, 1005 participants completed the questionnaire of whom 510 (50.8%) were men and 495 (49.3%) were women. The median age of respondents was 21 (interquartile range, 19-23). Eight hundred sixty-one (86.2%) participants reported ever having had sexual intercourse. Of these, 280 (32.5%) agreed to provide urine samples for *C trachomatis *testing. An additional two respondents' urine samples were inhibited on PCR, three who did not provide sufficient urine for analysis, and one respondent's sample was lost during shipping; we excluded these six from further analysis.

Table 1 shows the proportion of those agreeing to provide urine samples by socio-demographic and behavioural variables. In multivariate analysis, women had a significantly higher likelihood of providing a urine sample compared to men, as did those who did not use condoms at last intercourse.

**Table 1:** Factors associated with providing urine among those who had sexual intercourse

	Provided urine (%)	Base	**Unadjusted OR**^a ^ (95% CI)	Adjusted OR (95% CI)
**Total**	32.5	861		

**Gender**			p = 0.003	p = 0.004
Male	27.9	445	1.0	1.0
Female	37.5	416	1.55 (1.17-2.07)	1.53 (1.14-2.06)

**Age**			p = 0.044	p = 0.160
18-21	29.0	393	1.0	1.0
22-25	35.5	468	1.35 (1.00-1.80)	1.24 (0.92-1.67)

**Self-estimated socio-economic status**			p = 0.132	p = 0.074
Better than/equal to the others	31.9	832	1.0	1.0
Worse than the others	48.3	29	1.99 (0.94-4.17)	2.01 (0.93-4.31)

**Lived with both parents until 18 years of age**			p = 0.142	p = 0.252
Yes	31.8	746	1.0	1.0
No	38.9	108	1.37 (0.90-2.07)	1.29 (0.84-1.98)

**Got information about HIV/AIDS at school**			p = 0.170	p = 0.112
No	22.5	40	1.0	1.0
Yes	33.0	821	1.70 (0.80-3.62)	1.88 (0.86-4.1)

**Age of first sexual intercourse**			p = 0.273	
≤16	30.4	306	1.0	
≥17	34.1	546	1.18 (0.88-1.60)	

**Number of partners in the past 12 months**			p = 0.879	
0-1	32.2	549	1.0	
≥2	32.8	290	1.02 (0.76-1.39)	

**Lifetime number of partners**			p = 0.800	
0-1	30.8	185	1.0	
2-3	33.2	244	1.12 (0.74-1.68)	
≥4	33.6	393	1.14 (0.78-1.65)	

**Used condom at last intercourse**			p < 0.0001	p < 0.0001
Yes	24.8	407	1.0	1.0
No	39.4	454	1.97 (1.47-2.64)	1.95 (1.44-2.62)

**Concurrent partnerships ever**			p = 0.373	
No	33.5	656	1.0	
Yes	30.2	199	0.86 (0.61-1.21)	

^a ^OR = odds ratio; 95% CI = 95% confidence interval

^b ^p-values represent significance test for heterogeneity across the variable

Prevalence of *C trachomatis *infection among those who had sexual intercourse (n = 274) was 6.2% (95% CI 3.3%-9.1%) (Table 2). We investigated *C trachomatis *positivity by different determinants, such as gender, socio-economic and behavioural characteristics, but none of these was significantly associated with *C trachomatis *positivity at p < 0.05 level.

**Table 2:** Prevalence of *Chlamydia trachomatis *infection by demographic and behavioural variables^a^

	% (n)	Total number of participants in the subgroup
**Total among those who had intercourse**	6.2 (17)	274

**Gender**		p = 0.491
Male	7.3 (9)	123
Female	5.3 (8)	151

**Self-estimated socio-economic status**		p = 0.601^b^
Better than/equal to the others	6.2 (16)	260
Worse then the others	7.1 (1)	14

**Professional status**		p = 0.207 ^b^
Attending secondary school	3.5 (1)	29
University student	4.3 (4)	94
Employed	4.6 (5)	109
Unemployed	12.3 (7)	57

**Living with both parents until 18 years of age**		p = 0.150 ^b^
Yes	5.2 (12)	232
No	12.2 (5)	41

**Got information about HIV/AIDS at school**		p = 0.101 ^b^
No	22.2 (2)	9
Yes	5.7 (15)	265

**Age of first sexual intercourse**		p = 0.501
≤ 16	7.6 (7)	92
≥17	5.5 (10)	181

**Number of partners in the past 12 months**		p = 0.296
0-1	5.2 (9)	172
≥2	8.5 (8)	94

**Lifetime number of partners**		p = 0.175 ^b^
0-1	1.8 (1)	56
2-3	3.9 (3)	78
>4	8.5 (11)	130

**Used condom at last intercourse**		p = 0.551
Yes	5.1 (5)	99
No	6.9 (12)	175

**Concurrent partnerships ever**		p = 0.543^b^
No	5.6 (12)	214
Yes	8.3 (5)	60

^a^Among those who had sexual intercourse; ^b ^Fisher exact test

Sixty-nine (23.4%) participants called to obtain test results, and out of these, only four were diagnosed with *C trachomatis *infection. Those positive for infection were asked for their postal addresses when they called to ask for their test results. They were sent the test results by mail, and were instructed to seek treatment from gynaecologists or general practitioners.

## Discussion

This is the first general population-based survey on sexual behaviours in young adults in Croatia that included willingness to test for *C trachomatis*. The response rate for collecting urine specimens was 32.5%, and the prevalence of *C trachomatis *infection in sexually active individuals was 7.3% in men and 5.3% in women. Women, those who perceived their socio-economic status to be lower than average compared to their peers and those who did not use condoms at last intercourse were more likely to provide urine, possibly indicating an awareness of greater vulnerability towards STIs.

The main limitation in interpretation of determinants for *C trachomatis *prevalence is a low response rate to urine specimen collection. This could be due to a lack of awareness of young adults of the importance of screening for *C trachomatis*, or alternatively of inadequate skills of interviewers and operational difficulties in the field implementation of such studies. Urine samples were transported from participants' homes to the Counties Institutes of Public Health (there are 21 counties in Croatia), and from there to the "Dr. Fran Mihaljević" University Hospital for Infectious Diseases in Zagreb. Such operational difficulties in carrying out integrated bio-behavioural surveys of STIs have been described elsewhere [13,14].

The response rate achieved in our survey is similar to that achieved in population-based *C trachomatis *prevalence assessment surveys that used mail-delivered testing among young adults in Tartu, Estonia (34%), and the Netherlands (41%) [15,16]. In Estonia, prevalence of *C trachomatis *among women and men aged 18-35 years was 6.9% and 2.7%, respectively [15]. In the Netherlands, the prevalence of *C trachomatis *infection was 2.5% in women and 1.5% in men 15-29 years old [16]. In Eastern Europe, a household-based probability sample survey that estimated prevalence of infection with *C trachomatis *was carried out in Slovenia in 2001 among 18-49 year olds [8]. It found the prevalence of *C trachomatis *infection to be 4.1% among both men and women 18-24 years old, and the response rate to urine specimen collection was 50.9% among men and 60.0% among women. In Great Britain, prevalence of *C trachomatis *infection was 2.7% among men and 3.0% among women aged 18-24 in a household-based probability sample survey carried out in 2001 [9].

In the US, a number of household surveys have measured chlamydia prevalence. In three studies carried out amongst young adults, response rates of 61-81% were achieved [17-19]. The response rates in the studies carried out in the US are higher than in our study, possibly because of monetary and other incentives (food coupons) given for participating.

Compared to the European surveys that obtained similar response rates, the prevalence of *C trachomatis *found in this study is considerable, particularly in men. However, as the results suggest, those with higher levels of risk behaviours (not using condoms at last sexual intercourse) were more likely to provide urine samples, hence the prevalence observed in our study is likely to overestimate the true prevalence of *C trachomatis *in this population. Therefore, the prevalence of *C trachomatis *found in our study should not be generalized to the population aged 18-25 in Croatia due to greater willingness of those likely to be at higher risk to provide urine and the low response rate. Greater willingness of those with higher sexual risk behaviours to provide urine was also reported in the population-based survey in Great Britain [20].

Although findings were not statistically significant, in our study prevalence of *C trachomatis *was higher among those socially vulnerable, e.g., those who did not live with their parents up until the age of 18, unemployed and those with self-perceived lower socio-economic status. These findings provide strong evidence for the implementation of screening policies for *C trachomatis *in Croatia, and expanding access to STI prevention and control services to those socially and behaviourally vulnerable to this infection. Annual screening of all sexually active women younger than 25 years old is recommended in the United States, while in England, the National Chlamydia Screening Programme offers screening to men and women under the age of 25 years, either annually or when they change partners [21,22]. Such guidelines or screening programmes for *C trachomatis *infection do not exist in Croatia.

Only a small proportion of those who gave urine called to obtain their test results. This is in contrast to the results of the bio-behavioural survey among men who have sex with men done at the University Hospital for Infectious Diseases "Dr. Fran Mihaljevic" in Zagreb in 2006 when 78% of respondents came to collect their test results [23]. This might imply lower awareness among young adults of sexual health issues. Interviewers who also did urine specimen collection in our study reported that the vast majority of young adults whom they interviewed and asked to provide urine had low awareness of *C trachomatis *infection and were less likely to provide urine when parents were present at the household at the time of interviewing. They also reported that female and younger interviewers were more successful in obtaining urine specimens from respondents than their male and older counterparts.

In south-east Europe in particular, the absence of more comprehensive surveillance and over-reliance on routine case reporting contributes to perceptions that STIs are uncommon; this interferes with appropriate policy responses and presents a fundamental drawback in putting STI control higher on public health policy agendas. As mentioned earlier in the paper, *C trachomatis *case reporting rate in Croatia was 9.11/100,000 in 2007. Given the results of our survey, it appears that *C trachomatis *case reporting system substantially underestimates the burden of this infection. There is a pressing need to obtain a valid estimate of the burden of STIs, particularly curable bacterial STIs. Such estimates could lead to targeted responses, which would ideally be appropriate for and accessible to those at greatest risk, and likely driving disease transmission. The increased availability of nucleic acid amplification tests should be used to explore opportunistic screening and community-based testing in settings appropriate for young adults. In addition, such population-based surveys should be implemented in countries such as Croatia where STI case reporting systems substantially underestimates the true burden of disease.

In future surveys, efforts should be made to obtain a higher response rate. The bio-behavioural approach may be improved by adequate investments to ensure increased motivation of both interviewers and participants to provide samples and seek test results. In addition, efforts to increase knowledge about the consequences of untreated C *trachomatis *infection by carrying out targeted educational interventions might improve the uptake of home-based testing [24]. Raising awareness of STIs among young adults is important in countries like Croatia where systematic education on sexual health in primary and secondary schools in lacking. Small financial incentives for providing specimens in bio-behavioural surveys, in this case urine, might also increase participation rate [25].

## Conclusions

Population surveys that are household-based are a feasible option for obtaining population estimates of *C trachomatis *prevalence among young adults in Croatia.

In future, such population-based studies should have a large enough sample size to obtain better estimates in subgroups, and investigate further the gender and socio-economic differentials in prevalence of *C trachomatis*. In light of the high prevalence of *C trachomatis *observed in this study, there is a need for greater public health engagement into prevention, screening and diagnosis of this infection in Croatia.

## Competing interests

The authors declare that they have no competing interests.

## Authors' contributions

All authors (IB, IG, SZL, JIC, SBK, AS and JB) participated in the planning and conception of the research questions and the study design. IB, AS and JB conceptualised the study design and coordinated the study implementation. IG and SZL did the laboratory analysis of the samples. IB was responsible for analyzing the data. IB drafted the article, and all authors participated in interpreting the data and critically revising the manuscript. All authors read and approved the revised manuscript.

## Pre-publication history

The pre-publication history for this paper can be accessed here:

http://www.biomedcentral.com/1471-2458/11/230/prepub
